# The Impacts of Attitudes and Engagement on Electronic Word of Mouth (eWOM) of Mobile Sensor Computing Applications

**DOI:** 10.3390/s16030391

**Published:** 2016-03-18

**Authors:** Yu Zhao, Yide Liu, Ivan K. W. Lai, Hongfeng Zhang, Yi Zhang

**Affiliations:** 1 School of Business, Macau University of Science and Technology, Taipa, Macau 999078, China; 1209853gbm20024@student.must.edu.mo; 2 School of Business and Hospitality Management, Caritas Institute of Higher Education, Tseung Kwan O, New Territories, Hong Kong 999077, China; 3 Management Board Office, Macao Polytechnic Institute, Macau 999078, China; hfengzhang@ipm.edu.mo; 4 Department of Mathematics, Tongji University, Shanghai 200092, China; 08zhangyi@tongji.edu.cn

**Keywords:** mobile sensor computing, human–computer interaction (HCI), WeChat

## Abstract

As one of the latest revolutions in networking technology, social networks allow users to keep connected and exchange information. Driven by the rapid wireless technology development and diffusion of mobile devices, social networks experienced a tremendous change based on mobile sensor computing. More and more mobile sensor network applications have appeared with the emergence of a huge amount of users. Therefore, an in-depth discussion on the human–computer interaction (HCI) issues of mobile sensor computing is required. The target of this study is to extend the discussions on HCI by examining the relationships of users’ compound attitudes (*i.e.*, affective attitudes, cognitive attitude), engagement and electronic word of mouth (eWOM) behaviors in the context of mobile sensor computing. A conceptual model is developed, based on which, 313 valid questionnaires are collected. The research discusses the level of impact on the eWOM of mobile sensor computing by considering user-technology issues, including the compound attitude and engagement, which can bring valuable discussions on the HCI of mobile sensor computing in further study. Besides, we find that user engagement plays a mediating role between the user’s compound attitudes and eWOM. The research result can also help the mobile sensor computing industry to develop effective strategies and build strong consumer user—product (brand) relationships.

## 1. Introduction

As a two-way personal device, mobile helps users create and consume huge of data every day. Beyond sending/receiving SMS, mobile users could create a “virtual world” by using instant messengers (WhatsApp, WeChat, and other applications), wikis, blogs, social networking platforms and podcasts to access and share information. With the development of sensor computing recently, mobile users could get more benefit, including ubiquitous availability, fast response, and context-aware ability. Mobile sensor computing could help users share information while walking, driving or even sleeping can provide plenty of extremely useful applications, such as personal service (m-commerce, eHealthcare, exercise/fitness and safety), location-based services (nearby restaurants, bars, gas stations and ATMs), traffic service (accidents, congestion, road work) and vehicle service (VAENT) gathered through on board sensors. The next generation of mobile sensor computing will move forward on understanding external context, intelligent reminder mechanism, and network resources usage. In the future, users may get more external context data through mobile devices, such as temperature, sound, air pressure, surrounding users, and another context. Future mobile sensor computing could monitor the ubiquitous pervasive computing environment for new sensory data and build automatic decision algorithms to provide notices with less interruption. Standardized protocols and open APIs are also required to optimize network resources for different mobile sensor computing applications.

A typical example of mobile sensor computing application is WeChat. Beyond sharing feelings with friends through a social network, WeChat launched a new function named “Official Accounts”, which integrates the mobile sensor computing function for the business. By virtue of sensor computing, firms with WeChat Official Accounts could provide group buying functions and public service (traffic, weather, healthcare) announcements. The Official Account followers could order taxis and go shopping by location identification and use WeChat payment (NFC payment) services. TV programs/music nearby could be found for social networking. Real-time location could be shared for entertainment and safety. According to Ying Zhang, the vice president of product department of WeChat, there were more than 2 million Official Accounts in WeChat until November 2013, and these accounts processed over a hundred million items of interactive information every day [1].

Recent research shows that consumers have moved to mobile shopping from the in-store shopping through friends’ recommendations, customer reviews, or ratings via Web 2.0 platforms and location based services [2]. Since mobile sensor computing has become an important electronic commerce platform, firms have begun to treat this platform as their new “battlefield”. Meanwhile, in order to get public’s interest and reach the actual and potential clients, marketers are more willing to focus on mobile marketing than before. Besides, recommendations from trusted sources (e.g., members of the same network) are known to be the most useful and effective ways for product promotion [3].

Mobile sensor computing is powerful. However, users may not type or respond through mobile devices when they are not available—They may not be willing to spend their time on mobile information from which they do not get immediate benefit. Ways of improving the users’ using experience of the mobile sensor computing are worth considering. We need to find out what happens when a media innovation encounters the marketplace in the form of huge amounts of information available from mobile sensor computing. To answer these questions, a human—computer interaction (HCI) or human factors study is required, which will be good for design, evaluation and implementation of mobile sensor computing [4,5]. Besides, we need to find out the relationships among compound attitudes, customer engagement, and eWOM. Understanding the relationships among these constructs could not only help the marketers to develop effective mobile sensor computing marketing strategies and build strong consumer–brand (product) relationships, but also benefit future studies in HCI.

## 2. Literature Review, Research Gaps and Research Model

### 2.1. Attitudes

Based on the concept of human learned disposition, Kotler defined attitude as an expression of the individual personal evolution, an action tendency, and an emotional feeling towards some objects or ideas [6]. Bohner and Dickel argued attitude is an evaluation of an object of thought and it could be influenced by [7]. Some scholars have stated that attitude is a long-lasting assessment of the contextual elements [8]. Other authors (e.g., Schiffman and Kanuk [9]) considered attitude as a predisposition to behave with respect to a given object. According to the motivation and opportunity as determinants (MODE) model (Fazio [10]) and the meta-cognitive model (Petty, Briñol, and DeMarree [11]), attitude is treated as long-term memory structures.

Higgins indicated that attitude is more accessible after being constructed many times in similar situations [12]. Contrarily, Schwarz proposed a model to argue that attitudes are not constructed from enduring personal dispositions, but more like evaluative judgments which shaped in the situation similar to the current context [13]. Eagly and Chaiken presented an “umbrella definition” of attitude to embrace the critical elements of tendency, attitude object, and evaluation [14]. Cunningham *et al*. thought that attitudes are constructed from relatively stable representations [15].

In psychology, attitude can be understood as a particular cognitional process [6]. Previous social psychology literature indicated that attitude should conceptually separate into two dimensions: Affect and cognition [16,17,18]. Studies described cognitive component as the faith or knowledge a person holds toward things [19,20]. Therefore, a sense of fact statement in evaluation often comes with the attitude cognition, which suggests the agreement or refusal a person feel toward the attitudinal subjects.

In this study, attitude includes affective attitude (AA, users can obtain a mobile sensor computing application when they feel happy, positive, and good) and cognitive attitude (CA, users can obtain a mobile sensor computing application when they feel wise, beneficial, and valuable).

### 2.2. Engagement

The term ‘‘engagement’’ has been discussed in different fields, such as psychology, sociology, political science, and organizational behavior [21]. In the organizational behavior literature, the concept of engagement has been explored as a mean to explain organizational commitment and organizational citizenship behavior [22]. In the marketing and service literature, very few academic articles used the terms ‘‘engagement’’ prior to 2005 [23]. In contrast, the term “involvement” is more popular. In general terms, involvement refers to personal phenomena. Involvement is related to an individual’s needs, values, and self-concept, and it implicitly expresses the person’s beliefs and feelings about an object in a particular situation [24,25]. Involvement influences information searching, information processing, and decision making [26]. Brodie *et al.* distinguished the engagement from ‘‘involvement’’—The concepts of ‘‘involvement’’ or ‘‘participation’’ may be viewed as customer engagement antecedents, instead of dimensions [23]. Mollen and Wilson also thought “involvement” fails to reflect the notion of interactive experience [27]. Customer engagement is based on a customer’s co-creative experiences [28].

Pertaining to engagement contexts, Web 2.0 applications create a unique platform for users [29,30]. Bezjian-Avery *et al*. found that consumer engagement may be used to assess the effectiveness of interactive media advertising [31]. Hollebeek recognized the importance of customer engagement in the Web 2.0 applications, which could help to share information and value based on user bases [21]. Gambetti and Graffigna highlighted that media is one of the central roles of consumer engagement in maintaining customer - brand relationships [32]. Customer engagement has a positive effect on online social platform participation and word-of-mouth communication [28]. Customer engagement in the online social platform can be seen as a construct including vigor, absorption and dedication towards the online social platform, which is driven by involvement and social interaction [28].

### 2.3. eWOM

Arndt defined word-of-mouth (WOM) as informal communications among consumers on products or services [33]. Later, researchers made lots of effort to try to figure out the mechanism of WOM spreading. Early studies used psychological properties (e.g., customer satisfaction) to predict WOM behaviors [34]. The involvement and self-enhancement are also conducive to generating positive WOM [35].

The term electronic word-of-mouth (eWOM) has been defined as “any positive or negative statement made by potential, actual, or former customers about a product or company, which is made available to a multitude of people and institutions via the Internet” [36]. Recently, study regards eWOM as spreading behaviors by which consumers post their personal experiences (e.g., online review; arguments; recommendations) of specific products or services and generate convictive effects on the targeted receivers by using the internet [37].

Chu and Kim indicated that eWOM in Social Network Sites (SNSs) conceptually included three aspects: Opinion seeking, opinion giving and opinion passing [38]. When consumers made a purchase decision, some of them are more likely to search for information and advice from others because of opinion seeking behavior [39]. In contrast, the opinion leaders may cause a significant influence on others’ behavior and attitude by spreading their comments [40]. Dellarocas argued that under the online social context, opinion passing behavior could easily reach to the receivers since the multidirectional communications on the internet is quite a common thing [41]. Hence, Chu and Kim pointed out that opinion passing behavior is a supplement concept of eWOM in SNSs [38].

Since the late 1990s, the rapid proliferation of the internet has enabled consumers to spread their post-purchase experience through such online communications as email, website bulletin boards, news-groups, and blogs [42]. With the emergence of social networking commerce, there has been growing interests on searching and exchanging the eWOM [41]. Following this trend, Okazaki argued that WOM research should focus on ubiquitous media as both information seeker and source are likely to exchange information via mobile devices [43]. eWOM strongly influences the customer behaviors [37,38,44,45]. Varadarajan and Yadav pointed out four important changes that are occurring in the buying environment as a result of eWOM: Facilitating access to the type and amount of information; increasing ease of comparing and evaluating; improving the quality of information; organizing and structuring information [46]. eWOM has become increasingly popular with the rapid growth of availability and ubiquitous in mobile communication and firms have attempted to disseminate promotional campaigns via mobile internet channels [42,47]. In this study, eWOM included three aspects: Opinion seeking, opinion giving and opinion passing.

### 2.4. Research Gap, Research Model and Hypotheses

The preceding literature review reflects a substantial amount of research on the subjects of WeChat, attitude, customer engagement, and eWOM. Scholars have showed enormous enthusiasm in studying WeChat, the research topics including: The commercial potential of WeChat, CRM in WeChat, *etc*. However, most of the articles focused on practice, instead of a theory or empirical research. Researchers didn’t figure out the mechanism of HCI in WeChat till now. According to marketing literature, attitude is a predictor of consumers’ behavior, however, one of the major drawbacks of these studies is the failure to address how attitude influence customer engagement and eWOM behavior. Very little research has focused on the concept of customer mobile sensor computing engagement [21]. Little theory-guided research has been undertaken to understand the nature of customer engagement and eWOM in the specific context of mobile sensor computing [28]. Most studies regard eWOM as an antecedent of expectation, perception, and behavioral intention. In contract, not many scholars emphasize eWOM as an outcome variable in their conceptual frameworks, and the communication process and communication effectiveness of eWOM are still not clear. Hence, our study will endeavor to bridge these gaps by figuring out the relationships among attitude, customer engagement, and eWOM in the context of WeChat, which will be helpful for design and evaluate mobile sensor computing applications.

According to Saks, engagement is positively related to attitudes [22]. Numerous evidence demonstrated that attitudes influence both of the processing of information and behavior [7,48]. Calder and Malthouse indicated that engagement is ‘‘the sum of the motivational experiences’’ [49]. The experiences could be customer’s attitudes toward online social media platform [49]. Mollen and Wilson argued that online engagement is the customer’s cognitive and affective commitment to a computer-mediated brand value [27]. Overall, these pieces of evidence indicate that attitudes will affect customer engagement. Therefore, the following hypotheses are formulated to explore the relationships between attitude and customer engagement in the context of mobile sensor computing:

- H1a. Brand (product) related affective attitudes positively influences vigor
- H1b. Brand (product) related affective attitudes positively influences absorption
- H1c. Brand (product) related affective attitudes positively influences dedication
- H2a. Sensor computing platform related affective attitudes positively influences vigor
- H2b. Sensor computing platform related affective attitudes positively influences absorption
- H2c. Sensor computing platform related affective attitudes positively influences dedication
- H3a. Brand (product) related cognitive attitudes positively influences vigor
- H3b. Brand (product) related cognitive attitudes positively influences absorption
- H3c. Brand (product) related cognitive attitudes positively influences dedication
- H4a. Sensor computing platform related cognitive attitudes positively influences vigor
- H4b. Sensor computing platform related cognitive attitudes positively influences absorption
- H4c. Sensor computing platform related cognitive attitudes positively influences dedication

A consumer will make behavioral (e.g., eWOM) intention directly to a specific brand or product. A positive attitude will reflect in a positive evaluation of the brand or product [6]. Studies pointed out that engaged customers may experience confidence in the brand [50,51,52]. Saks argued that engagement positively related to individuals’ intentions and behaviors [22]. Social judgment theory assumed that people would judge and assimilate new information base on existing feelings [53]. Attitude and contextual information are correlated positively based on assimilation effect [13]. Thus, following hypotheses are formulated to figure out the relationships between attitude and eWOM in the context of mobile sensor computing:

- H5a. Brand (product) related affective attitudes positively influences opinion seeking
- H5b. Brand (product) related affective attitudes positively influences opinion giving
- H5c. Brand (product) related affective attitudes positively influences opinion passing
- H6a. Sensor computing platform related affective attitudes positively influences opinion seeking
- H6b. Sensor computing platform related affective attitudes positively influences opinion giving
- H6c. Sensor computing platform related affective attitudes positively influences opinion passing
- H7a. Brand (product) related cognitive attitudes positively influences opinion seeking
- H7b. Brand (product) related cognitive attitudes positively influences opinion giving
- H7c. Brand (product) related cognitive attitudes positively influences opinion passing
- H8a. Sensor computing platform related cognitive attitudes positively influences opinion seeking
- H8b. Sensor computing platform related cognitive attitudes positively influences opinion giving
- H8c. Sensor computing platform related cognitive attitudes positively influences opinion passing

Brodie *et al.* identified that engaged customers play a key role in providing referrals and recommendations for specific products or services [23]. The customer should not only be satisfied with the product but also be willing to promote the product [54]. eWOM could be considered as one of these promotion behaviors. Beside, Vivek *et al*. suggested that customer is positively associated with an individual’s WOM activity [55]. Bowden argued that emotion could drive WOM recommendation [56]. Chu and Kim indicated that the consumer- social network relationships should play a key role in shaping eWOM [38]. Furthermore, if a customer is willing to add information to an online social platform, he or she will have a higher propensity to participate in an online social platform, as well as to spread eWOM [28]. From these perspectives, it is reasonable to argue that customer engagement will affect eWOM. Hence, following hypotheses are formulated to explore the relationships between customer engagement and eWOM in the context of mobile sensor computing:

- H9a. Vigor positively influences opinion seeking
- H9b. Vigor positively influences opinion giving
- H9c. Vigor positively influences opinion passing
- H10a. Absorption positively influences opinion seeking
- H10b Absorption positively influences opinion giving
- H10c. Absorption positively influences opinion passing
- H11a. Dedication positively influences opinion seeking
- H11b. Dedication positively influences opinion giving
- H11c. Dedication positively influences opinion passing

To this point, we have argued affective attitude and cognitive attitude will guide the processing of information and influence behavior. Indeed, researchers indicated that customer engagement may be manifested cognitively, affectively, behaviorally, or socially [55]. Hence, we argued here that customer engagement plays an important role in explaining the relationships among attitude and eWOM. In another word, we have implicitly described a model in which customer engagement mediates relationships between compound attitudes and eWOM behavior. Thus, we posit the following hypothesis:

- H12. Customer engagement mediates the relationship between compound attitudes and eWOM behavior

**Figure 1:**
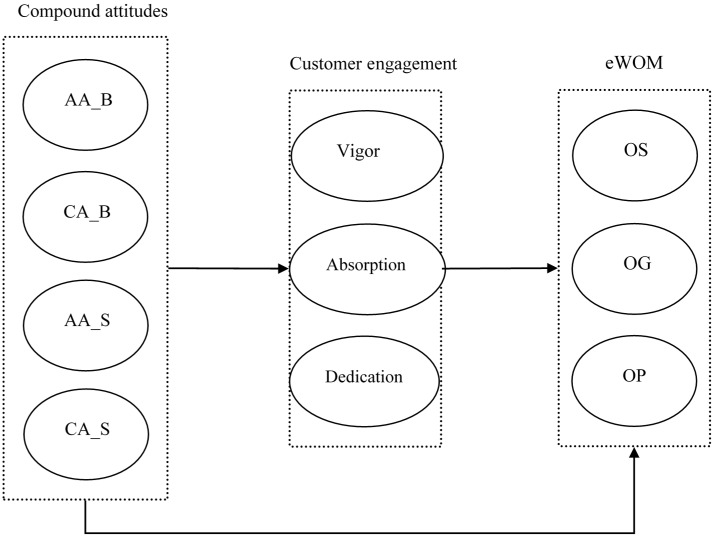
Research model. Note. AA_B = brand (product) related affective attitudes; AA_S = sensor computing platform related affective attitudes; CA_B = brand (product) related cognitive attitudes; CA_S = sensor computing platform related cognitive attitudes; OS = opinion seeking; OG = opinion giving; OP = opinion passing.

Figure 1 shows the research model based on the hypothesis that we have discussed.

## 3. Research Method

### 3.1. Measures of Constructs

Attitudes, engagement, and eWOM have been widely discussed in the literature. The choice of scales for our study constructs has therefore been based on the findings of previous publications then adapted to the context of our study. Structured questionnaires comprising 33 items were used to measure the 10 constructs mentioned in the research model. At the beginning of the survey, respondents were first asked to recall their feelings of a most impressive or attractive Official Accounts from WeChat. Next, participants rated their own brand (product) related affective attitude, mobile sensor computing platform related affective attitude, brand (product) related cognitive attitude, mobile sensor computing platform related cognitive attitude, vigor, absorption, dedication, opinion seeking, opinion giving, and opinion passing of the Official Accounts by using a five-point Likert scale that ranged from “strong disagree” (1) to “strong agree” (5).

In-depth interview was conducted among a small group (12 students) of heavy users of WeChat to revise and adjust the scale of the research constructs in the context of WeChat. The students have been asked three open-end questions: 1. The user experience of WeChat; 2. The user experience of official accounts; 3. The opinion of WeChat marketing. All the responses and answers were noted during the interview. The measured items are listed in Table 1.

### 3.2. Data Collection

In order to test the research model, we collected data from a convenience sample of university students from Macau University of Science and Technology (MUST) by using a structured questionnaire. The university students were chosen as our research sample as college students were deemed as the critical SNS user [58].

The data collection procedure comprised two stages. First, we conducted a pilot study to pretest the survey instrument. During the pilot study stage, we formed the questionnaire base on the research theme and distributed to the university students. A total of 23 responses were collected in this stage. The results of the pilot test have been used for revising and refining the questions. After pilot test stage, we carried out the formal research by distributed the revised questionnaire. The same as the pretest stage, revised questionnaire were distributed to the university students from MUST. The survey was administered in the campus over a 4-week period and students from MUST were solicited for participation. We obtained responses from a total number of 313 students, resulting in a response rate of 86.9%.

## 4. Data Analyses and Results

### 4.1. Descriptive Analysis

The results of the descriptive analysis are presented in Table 2. On average, respondents were 20 year-old: 56.9% of them were female and 43.1% were male. As our sample was collected from university students, participants have all received good education: 78.3% of them had college or equal level education experience and 21.7% were postgraduate students. All of the respondents had experience in using WeChat: 42.5% of them used WeChat for more than 2 years and 44.7% of the respondents claimed they used WeChat for more than 3 hours per day.

### 4.2. Reliability and Construct Validity

We used Cronbach’s alpha to measure the reliability of our research constructs: Brand (product) related affective attitude, Brand (product) related cognitive attitude, Sensor computing platform related affective attitude, Sensor computing platform related cognitive attitude, Vigor, Absorption, Dedication, Opinion Seeking, Opinion Giving, Opinion Passing. As shown in Table 3, all Cronbach’s alpha of the constructs were exceeded Nunnally’s recommended benchmark (α = 0.703, α = 0.760, α = 0.845, α = 0.715, α = 0.734, α = 0.744, α = 0.721, α = 0.764, α = 0.741, α = 0.716, respectively). These results of the test represents all of the constructs in our research have a high level of internal consistency reliability within consistent and stable items.

Before conducting the factor analysis, each construct of the study was assessed for validity (from Table 4, Table 5, Table 6 and Table 7): Affective attitudes, affective attitudes, engagement and eWOM have good construct validity.

### 4.3. Correlation Analysis

Table 8 reports correlations among all research constructs and control variables. Almost all the constructs were positively associated with each other. However, there were no statistical significant connections between some constructs (*i.e.*, sensor computing platform related affective attitudes and absorption; sensor computing platform related cognitive attitudes and absorption; sensor computing platform related cognitive attitudes and opinion seeking; absorption and opinion seeking).

### 4.4. Factors Analysis and Mediation Effect Analysis

Normed Chi-square (*i.e.*, χ^2^/d*f*), incremental fit indexes (e.g., CFI; NFI; TLI) and absolute fit indexes (e.g., RMSEA; GFI; AGFI) were chose to measure the fitness of our research model. According to the acceptable thresholds of Fit indexes, we suggested that our model fits the data fairly well and can be used to conduct hypothesis tests (Table 9).

The parameter estimate statistics of our research model are presented in Table 10. H1a, H1b, and H1c assumed that brand (product) related affective attitudes directly and positively influences vigor, absorption, and dedication, respectively. The paths from brand (product) related affective attitudes to vigor, absorption and dedication were positive and statistically significant (standardized β = 0.209, *p* < 0.01; standardized β = 0.162, *p* < 0.05; standardized β = 0.258, *p* < 0.01; respectively). Thus, H1a, H1b, and H1c were supported by the data.

H2a, H2b, and H2c were also supported, as the paths to vigor, absorption and dedication from sensor computing platform related affective attitudes were statistically significant (standardized β = 0.379, *p* < 0.001; standardized β = 0.399, *p* < 0.001; standardized β = 0.338, *p* < 0.001; respectively). Consequently, mobile sensor computing platform related affective attitudes have a direct and positive influence on vigor, absorption and dedication.

In H3a, it was presumed that brand (product) related cognitive attitudes directly and positively influences vigor. This path was statistically significant (standardized β = 0.252, *p* < 0.001). The association between brand (product) related cognitive attitude and absorption (standardized β = 0.256, *p* < 0.001) was also supported by the data. By contrast, the path from Brand (product) related cognitive attitude to the dedication, which was hypothesized in H3c, was not statistically significant (standardized β = 0.115, *p* = 0.108). Hence, H3c was unsupported.

The standardized path estimates provided support for H4a, H4b, and H4c. Therefore, mobile sensor computing platform related cognitive attitude has a direct and positive influence on vigor, absorption and dedication.

H5a, H5a, and H5c assumed that brand (product) related affective attitudes directly and positively influences opinion seeking, opinion giving, and opinion passing, respectively. The paths from brand (product) related affective attitudes to opinion seeking and opinion giving were positive and statistically significant (standardized β = 0.511, *p* < 0.001; standardized β = 0.485, *p* < 0.001; respectively). Thus, H5a and H5b were both supported by the data. By contrast, the path from a brand (product) related affective attitudes to opinion passing, which was hypothesized in H5c, was not statistically significant (Standardized β = 0.247, *p* = 0.450). This result was contrary to the expectation.

In H6a, it was presumed that sensor computing platform related affective attitudes directly and positively influences opinion seeking. This path was not statistically significant (standardized β = 0.400, *p* = 0.123). Therefore, H6a was not supported by the data. By contrast, the paths from mobile sensor computing platform related affective attitudes to opinion giving and opinion passing, which were hypothesized in H6b and H6c, were statistically significant (standardized β = 0.716, *p* < 0.001; standardized β = 0.606, *p* < 0.001, respectively).

H7a, H7b, and H7c assumed that brand (product) related cognitive attitudes directly and positively influences opinion seeking, opinion giving, and opinion passing, respectively. The paths from brand (product) related cognitive attitudes to opinion seeking, opinion giving, and opinion passing, were positive and statistically significant (standardized β = 0.392, *p* < 0.001; standardized β = 0.402, *p* < 0.001; standardized β = 0.452, *p* < 0.001, respectively). Thus, H7a, H7b, and H7c were supported by the data.

H8a, H8b, and H8c were also supported. Therefore, sensor computing platform related cognitive attitudes has a direct and positive influence on opinion seeking, opinion giving, and opinion passing.

In H9a, it was presumed that vigor directly and positively influences opinion seeking. This path was statistically significant (standardized β = 0.260, *p* < 0.05). The same as H9a, H9c was also supported by the data. By contrast, the path from Vigor to opinion giving, which was hypothesized in H9b, was not statistically significant (standardized β = 0.174, *p* = 0.139).

H10a, H10b, and H10c assumed that absorption directly and positively influences opinion seeking, opinion giving, and opinion passing, respectively. The paths from absorption to opinion seeking, opinion giving, and opinion passing were positive and statistically significant (standardized β = 0.435, *p* < 0.001; standardized β = 0.703, *p* < 0.001; standardized β = 0.600, *p* < 0.001, respectively). Accordingly, H10a, H10b, and H10c were supported by the data.

In H11a, it was presumed that dedication directly and positively influences opinion seeking. This path was statistically significant (standardized β = 0.408, *p* < 0.001). H11c was also supported by the data. By contrast, the path from dedication to opinion giving, which was hypothesized in H11b, was not statistically significant (standardized β = 0.081, *p* = 0.398). H3c was unsupported.

For mediation effect analysis, we tested the total effects of the independent variables (*i.e.*, the dimensions of compound attitudes) on the dependent variables (*i.e.*, the dimensions of eWOM). The results of standardized total effect estimate and significance test are shown in Table 11 and Table 12 respectively. As can be seen from Table 12, all standardized total effect estimates are statistically significant except the path from a brand (product) related affective attitudes to opinion passing and the path from sensor computing platform related affective attitudes to opinion seeking. According to [28,38], dimensions of customer engagement (*i.e.*, vigor, absorption, and dedication) neither mediate the relationship between brand (product) related affective attitudes and opinion passing nor the relationship between sensor computing platform related affective attitudes and opinion seeking.

The direct effects of compound attitudes (*i.e.*, sensor computing platform related cognitive attitudes, brand (product) related cognitive attitudes, sensor computing platform related affective, brand (product) related affective attitudes) on customer engagement and customer engagement on eWOM were tested in the level of dimension respectively. As shown in Table 13, all standardized direct effect estimates are statistically significant except the path from a brand (product) related cognitive attitudes to dedication, the path from the dedication to opinion giving, and the path from vigor to opinion passing.

Table 14 reports the indirect effect estimates of compound attitudes on eWOM in the level of dimension respectively. Meanwhile, the significance of these estimates is presented in Table 15. To our surprise, all of the indirect effect estimates are not statistically significant. Accordingly, we argued that:

Vigor fully mediates the relationship between brand (product) related affective attitudes and opinion seeking;

Vigor fully mediates the relationship between sensor computing platform related affective attitudes and opinion giving;

Vigor fully mediates the relationship between brand (product) related cognitive attitudes and opinion seeking;

Vigor fully mediates the relationship between brand (product) related cognitive attitudes and opinion giving;

Vigor fully mediates the relationship between sensor computing platform related cognitive attitudes and opinion seeking;

Vigor fully mediates the relationship between sensor computing platform related cognitive attitudes and opinion giving;

Absorption fully mediates the relationship between brand (product) related affective attitudes and opinion seeking;

Absorption fully mediates the relationship between brand (product) related affective attitudes and opinion giving;

Absorption fully mediates the relationship between sensor computing platform related affective attitudes and opinion giving;

Absorption fully mediates the relationship between sensor computing platform related affective attitudes and opinion passing;

Absorption fully mediates the relationship between brand (product) related cognitive attitudes and opinion seeking;

Absorption fully mediates the relationship between brand (product) related cognitive attitudes and opinion giving;

Absorption fully mediates the relationship between brand (product) related cognitive attitudes and opinion passing;

Absorption fully mediates the relationship between sensor computing platform related cognitive attitudes and opinion seeking;

Absorption fully mediates the relationship between sensor computing platform related cognitive attitudes and opinion giving;

Absorption fully mediates the relationship between sensor computing platform related cognitive attitudes and opinion passing;

Dedication fully mediates the relationship between brand (product) related affective attitudes and opinion seeking;

Dedication fully mediates the relationship between sensor computing platform related affective attitudes and opinion passing;

Dedication fully mediates the relationship between sensor computing platform related cognitive attitudes and opinion seeking;

Dedication fully mediates the relationship between sensor computing platform related cognitive attitudes and opinion passing.

According to Table 16, the standardized regression coefficient and the standard error of the path from brand (product) related cognitive attitudes to dedication are 0.115 and 0.033. Meanwhile, the standardized regression coefficient and the standard error of the path from dedication to opinion passing are 0.596 and 0.037. Hence, dedication mediates the relationship between brand (product) related cognitive attitudes and opinion passing with a small mediated effect.

Similarly, we demonstrated that dedication mediates the relationship between brand (product) related cognitive attitudes and opinion giving (z = 3.329, *p* < 0.05, small mediated effect); dedication mediates the relationship between brand (product) related cognitive attitudes and opinion seeking (z = 3.420, *p* < 0.05, small mediated effect); dedication mediates the relationship between sensor computing platform related cognitive attitudes and opinion giving (z = 10.573, *p* < 0.05, large mediated effect); dedication mediates the relationship between sensor computing platform related affective attitudes and opinion giving (z = 7.417, *p* < 0.05, medium mediated effect); dedication mediates the relationship between brand (product) related affective attitudes and opinion giving (z = 9.137, *p* < 0.05, large mediated effect); vigor mediates the relationship between sensor computing platform related affective attitudes and opinion passing (z = 10.743, *p* < 0.05, large mediated effect); vigor mediates the relationship between brand (product) related affective attitudes and opinion passing (z = 10.460, *p* < 0.05, large mediated effect); vigor mediates the relationship between sensor computing platform related cognitive attitudes and opinion passing (z = 8.494, *p* < 0.05, large mediated effect); vigor mediates the relationship between brand (product) related cognitive attitudes and opinion passing (z = 10.720, *p* < 0.05, large mediated effect).

Combining the mediating effect analyses above, we inferred that H12 (Customer engagement mediates the relationship between compound attitudes and eWOM behavior was partially supported by the data.

**Figure 2:**
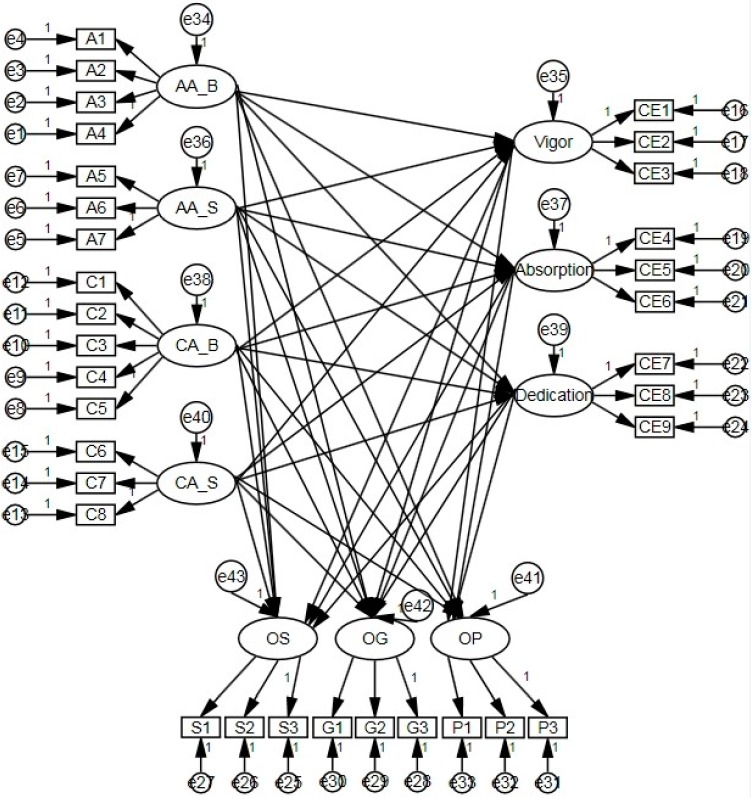
Path diagram of the proposed model.

**Figure 3:**
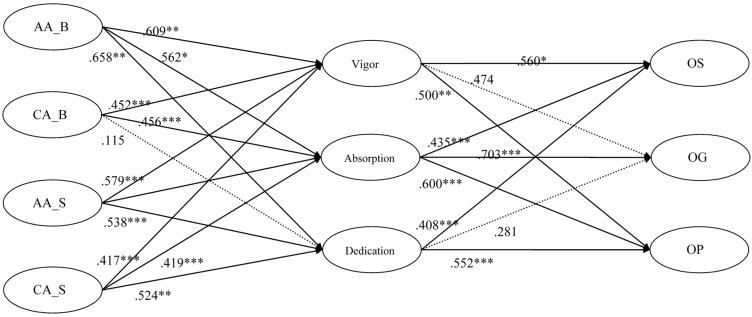
Research model with standardized path coefficients (Part 1).Note. * *p* < 0.05, ** *p* < 0.01, *** *p* < 0.001; AA_B = brand (product) related affective attitudes; AA_S = sensor computing platform related affective attitudes; CA_B = brand (product) related cognitive attitudes, CA_S = sensor computing platform related cognitive attitudes; OS = opinion seeking, OG = opinion giving; OP = opinion passing.

**Figure 4:**
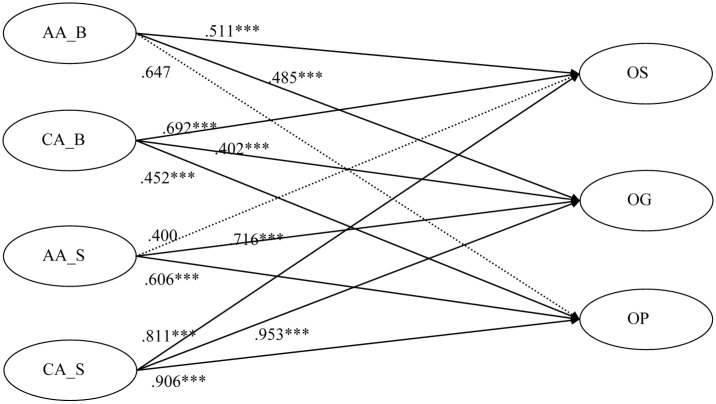
Research model with standardized path coefficients (Part 2). Note. * *p* < 0.05, ** *p* < 0.01, *** *p* < 0.001; AA_B = brand (product) related affective attitudes; AA_S = sensor computing platform related affective attitudes; CA_B = brand (product) related cognitive attitudes; CA_S = sensor computing platform related cognitive attitudes; OS = opinion seeking; OG = opinion giving; OP = opinion passing.

The path diagram of the propose model and research model with standardized path coefficients are shown from Figure 2, Figure 3 and Figure 4.

## 5. Discussion

### 5.1. Theoretical Contributions

First, this research examined the relationships among compound attitude, engagement, and eWOM in the context of mobile sensor computing application. Traditionally, most researches treat eWOM as an antecedent of behavioral intention. We analyzed the relationships among compound attitude, engagement, and eWOM on different dimensional levels. Our research indicated that Brand (product) related affective attitudes positively influences vigor, absorption, dedication, opinion giving, and opinion seeking; sensor computing platform related affective attitudes is positively associated with vigor, absorption, dedication, opinion giving, and opinion passing; Brand (product) related cognitive attitude positively influences vigor, absorption, opinion seeking, opinion giving, and opinion passing; sensor computing platform related cognitive attitudes is positively associated with vigor, absorption, dedication, opinion seeking, opinion giving, and opinion passing; vigor positively influences opinion seeking and opinion passing; absorption is positively associated with opinion seeking, opinion giving, and opinion passing; dedication positively influences opinion seeking and opinion passing. Besides, we also found that the customer engagement partially mediates the relationship between compound attitudes and eWOM behaviors in the context of WeChat. Further, the results of moderating effect analyses indicated that gender has interaction effect on brand (product) related affective attitudes, social media platform related cognitive attitudes and opinion passing; education background has interaction effect on dedication and opinion passing; time of usage (total) has interaction effect on social media platform related affective attitudes, social media platform related cognitive attitudes, dedication and opinion passing; time of usage (daily) has interaction effect on social media platform related affective attitudes, social media platform related cognitive attitudes opinion giving, and opinion passing.

Second, our study enhanced the understanding of compound attitudes, customer engagement, and eWOM behaviors by delineating the eWOM process in WeChat. We empirically investigated the customer engagement as an important antecedent for eWOM behaviors in the context of mobile sensor computing, which is a lack of empirical evidence before. Furthermore, the empirical evidence indicated that compound attitudes which consist of brand-related attitudes and mobile sensor computing related attitudes are convictive to predict customer engagement behavior. Accordingly, our conceptual model is representative in the emerging mobile sensor computing platforms.

Theory of reasoned action, social cognitive theory, and theory of planned behavior model indicated that attitudes directly link to behavior intention or behaviors [59]. Our research reinforced these theories by empirically demonstrating the relationships between compound attitude and eWOM. Furthermore, we found that Brand (product) related affective attitude are not positively associated with opinion passing. We also found that sensor computing platform related affective attitude are not positively associated opinion seeking in come in the context of mobile sensor computing.

### 5.2. Implications for Practices

Mangold and Faulds argued that sensor computing plays a hybrid role in online business, as it enables companies to produce a unified consumer-centric advertising message to connect with their customers [60,61]. In contrast, eWOM in the context of sensor computing allows consumers to communicate each other when giving information in WeChat. When giving information in WeChat, consumers tend to share their product experience with all their contacts; these communications may also play a critical role in IMC. Our study suggested that in order to shape positive eWOM, a marketer should pay attention on the consumers’ attitude toward the brand (product) and try to engage them in the mobile sensor computing platforms.

This research not only enriches the theoretical knowledge about the determinant factors of eWOM in social media, but also helps IMC marketers to develop effective social media marketing strategies and build strong consumer–brand (product) relationships. We found that effort in building good customer engagement in mobile sensor computing can drive positive eWOM behavior. Since WeChat provides an efficient channel for building these relationships, marketers should try to encourage the users of WeChat to engage in their Official Accounts and spread positive eWOM regarding selected brands or products.

Our research found that compound attitudes will positively influence customer engagement in mobile sensor computing platform. A marketer should make efforts on shaping customer’s attitudes. In the context of Wechat, these efforts should be a target in building an attractive and friendly official account. Since customer engagement will positively influence customer’s eWOM behavior, affirmative and valuable eWOM behavior for a brand (product) are expected if a customer shows positive attitudes on engaging the social media. Empirical evidence from our research demonstrated that consumers will show opinion seeking, opinion giving opinion passing in the context of social media. As mentioned before, eWOM may not directly link to profit which marketer expected, but eWOM can affect the sales and consumers’ decision-making processes. Beside, Amblee and Bui indicated that eWOM can be used to convey the reputation of the product, the reputation of the brand, and the reputation of complementary goods [2,62,63,64]. By affecting the reputation of a brand (product), eWOM has a great influence on marketing.

Managers have been interested in customer engagement for about a decade. A large number of companies are providing platforms to get customers to come to their websites and purchase. However, companies are not sure where or how to target their efforts [55]. This paper suggests that the marketer should focus on both existing customers and potential customers’ compound attitude. Endeavor into customer brand (product) engagement on mobile sensor computing platforms may also help marketers to improve customer relationships.

### 5.3. Limitations and Further Studies

Our research sample was collected from both undergraduates and postgraduates of MUST in Macau, since college students represent the majority of SNS and mobile sensor computing platform users [38,65]. However, the country-specific factors cannot be ignored. Hence, future studies should discuss eWOM communication in mobile sensor computing varies across generations and regions.

Because of the limited time and resource, the causal relationships among the variables are still not crystal clear. Future studies should consider other factors that can lead to eWOM communication in mobile sensor computing and find out more antecedents which may influence customer engagement. In order to reduce the bias, further studies should conduct the researches through different types of mobile sensor computing platforms.

## Figures and Tables

**Table 1:** Scale of Compound Attitudes, Engagement and eWOM in WeChat.

Construct	Items	Source
Brand (product) related affective attitude	1. Regarding the use of the Official Accounts to access reviews about brand (product) evaluation, I feel very happy.	Shih *et al.* [37]
	2. Regarding the use of the Official Accounts to access reviews about brand (product) evaluation, I feel very positive.	
	3. I’m very like to use the Official Accounts to access reviews about brand (product) evaluation.	
	4. The Official Accounts is very attractive to me.	
Brand (product) related cognitive attitude	1. Regarding the use of the Official Accounts to access reviews about brand (product) evaluation, I feel very wise.	Shih *et al.* [37]
	2. Regarding the use of the Official Accounts to access reviews about brand (product) evaluation, I feel very beneficial.	
	3. Regarding the use of the Official Accounts to access reviews about brand (product) evaluation, I feel very valuable.	
	4. Regarding the use of the Official Accounts to access reviews about brand (product) evaluation, I feel very useful.	
	5. Regarding the use of the Official Accounts to access reviews about brand (product) evaluation, I feel very favorable.	
Mobile sensor computing platform related affective attitude	1. Using WeChat makes me feel happy.	Yang and Yoo [57]
	2. Using WeChat makes me feel positive.	
	3. Using WeChat makes me feel good.	
Mobile sensor computing platform related cognitive attitude	1. Using WeChat makes me feel wise.	Yang and Yoo [57]
	2. Using WeChat makes me feel beneficial.	
	3. Using WeChat makes me feel valuable.	
Vigor	1. I can continue using WeChat for very long periods at a time.	Cheung *et al.* [28]
	2. I feel vigorous when I am using WeChat.	
	3. I devote a lot of energy to WeChat.	
Absorption	1. I am rarely distracted when using WeChat.	Cheung *et al.* [28]
	2. My mind is focused when using WeChat.	
	3. I pay a lot of attention to WeChat.	
Dedication	1. I am enthusiastic in WeChat.	Cheung *et al.* [28]
	2. I found WeChat full of meaning and purpose.	
	3. I am interested in WeChat.	
Opinion Seeking	1. When I consider new products, I ask my contacts on WeChat for advice.	Chu and Kim [38]
	2. I like to get my contacts’ opinions on WeChat before I buy new products.	
	3. I feel more comfortable choosing products when I have gotten my contacts’ opinions on WeChat.	
Opinion Giving	1. I often persuade my contacts on WeChat to buy products that I like.	Chu and Kim [38]
	2. My contacts on WeChat pick their products based on what I have told them.	
	3. On WeChat, I often influence my contacts’ opinions about products.	
Opinion Passing	1. When I receive product related information or opinion from a friend, I will pass it along to my other contacts on WeChat.	Chu and Kim [38]
	2. On WeChat, I like to pass along interesting information about products from one group of my contacts on my “friends” list to another.	
	3. I tend to pass along my contacts’ positive reviews of products to other contacts on WeChat.	

**Table 2:** Descriptive statistics.

Item	Number	Percent
Gender		
Male	135	43.1%
Female	178	56.9%
Education background		
College	245	78.3%
Postgraduate or above	68	21.7%
Time usage of WeChat		
Less than 3 months	7	2.2%
3 months to 6 months	13	4.2%
7 months to 1 year	51	16.3%
1 year to 2 years	109	34.8%
More than 2 years	133	42.5%
Daily time usage of WeChat				
Less than 30 min per day	23	7.3%
30 min to 1 h per day	56	17.9%
1 to 2 h per day	53	16.9%
2 to 3 h per day	41	13.1%
More than 3 h per day	140	44.7%

**Table 3:** Reliability of the research constructs.

Construct	Item	Cronbach’s Alpha
Brand (product) related affective attitude	4	0.703
Brand (product) related cognitive attitude	5	0.760
Sensor computing platform related affective attitude	3	0.845
Sensor computing platform related cognitive attitude	3	0.715
Vigor	3	0.734
Absorption	3	0.744
Dedication	3	0.721
Opinion Seeking	3	0.764
Opinion Giving	3	0.741
Opinion Passing	3	0.716

**Table 4:** Rotated component matrix of affective attitudes.

	Component 1	Component 2
AA_B1	0.118	**0.792**
AA_B2	0.165	**0.738**
AA_B3	0.022	**0.697**
AA_B4	0.129	**0.658**
AA_S1	**0.855**	0.063
AA_S2	**0.890**	0.125
AA_S3	**0.847**	0.197

*Note*. Extraction Method: Principal Component Analysis. Rotation Method: Varimax with Kaiser Normalization. Rotation converged in three iterations.

**Table 5:** Rotated component matrix of cognitive affective attitudes.

	Component 1	Component 2
CA_B1	**0.702**	0.200
CA_B2	**0.733**	−0.202
CA_B3	**0.681**	0.024
CA_B4	**0.7** **23**	0.319
CA_B5	**0.802**	0.066
CA_S1	0.035	**0.880**
CA_S2	−0.024	**0.914**
CA_S3	0.151	**0.808**

*Note*. Extraction Method: Principal Component Analysis. Rotation Method: Varimax with Kaiser Normalization. Rotation converged in three iterations.

**Table 6:** Rotated component matrix of customer engagement.

	Component 1	Component 2	Component 3
Vigor1	**0.834**	0.072	0.184
Vigor2	**0.823**	0.228	0.053
Vigor3	**0.7** **45**	0.112	0.257
Absorption1	0.258	**0.806**	−0.143
Absorption2	0.329	**0.767**	0.138
Absorption3	−0.132	**0.800**	0.295
Dedication1	0.443	−0.019	**0.640**
Dedication2	−0.009	0.120	**0.844**
Dedication3	0.340	0.114	**0.726**

*Note*. Extraction Method: Principal Component Analysis. Rotation Method: Varimax with Kaiser Normalization. Rotation converged in three iterations.

**Table 7:** Rotated component matrix of eWOM.

	Component 1	Component 2	Component 3
OS1	0.108	**0.720**	0.421
OS2	0.254	**0.835**	0.100
OS3	0.230	**0.784**	−0.026
OG1	**0.802**	0.134	0.291
OG2	**0.814**	0.285	0.134
OG3	**0.606**	0.246	0.215
OP1	0.524	0.102	**0.645**
OP2	0.271	0.024	**0.761**
OP3	0.124	0.206	**0.817**

*Note*. Extraction Method: Principal Component Analysis. Rotation Method: Varimax with Kaiser Normalization. Rotation converged in three iterations.

**Table 8:** Means, standard deviations, and correlations of the research variables.

Variable	Mean	S.D.	1	2	3	4	5	6	7	8	9	10	11	12	13	14
1. Age	20.796	2.056														
2. Gender	0.431	0.496	0.112 *													
3. Education	3.217	0.413	0.483 **	0.104												
4. TUW	4.112	0.973	0.528 **	−0.054	0.442 **											
5. DTUW	3.700	1.382	0.076	−0.297 **	0.160 **	0.402 **										
6. AA_B	3.652	0.605	−0.043	−0.128 *	−0.017	0.102	0.122 *									
7. AA_S	3.705	0.810	−0.018	−0.102	−0.041	0.241 **	0.393 **	0.288 **								
8. CA_B	3.452	0.570	−0.072	−0.048	−0.021	0.031	0.027	0.492 **	0.190 **							
9. CA_S	4.158	0.670	0.023	−0.125 *	−0.012	0.230 **	0.336 **	0.217 **	0.710 **	0.178 **						
10. Vigor	3.308	0.683	0.034	0.055	−0.041	0.064	0.051	0.360 **	0.234 **	0.287 **	0.122 *					
11. Absorption	3.254	0.718	−0.028	0.025	−0.013	0.004	−0.022	0.268 **	0.096	0.296 **	0.106	0.346 **				
12. Dedication	3.470	0.627	−0.002	−0.011	−0.119 *	0.071	−0.045	0.315 **	0.154 **	0.207 **	0.155 **	0.440 **	0.258 **			
13. OS	2.895	0.721	0.024	0.017	−0.020	−0.041	0.046	0.350 **	0.144 *	0.298 **	0.032	0.265 **	0.110	0.200 **		
14. OG	3.230	0.633	−0.038	−0.072	−0.032	0.083	0.173 **	0.432 **	0.263 **	0.264 **	0.153 **	0.386 **	0.115 *	0.332 **	0.516 **	
15. OP	3.246	0.681	0.077	−0.135 *	0.155 **	0.144*	0.204 **	0.259 **	0.173 **	0.309 **	0.138 **	0.384 **	0.244 **	0.284 **	0.413 **	0.600 **

Note. *n* = 313, * *p* < 0.05, ** *p* < 0.01; TUW = time of usage (total); DTUW = time of usage (daily); AA_B = brand (product) related affective attitudes; AA_S = sensor computing platform related affective attitudes; CA_B = brand (product) related cognitive attitudes; CA_S = sensor computing platform related cognitive attitudes, OS = opinion seeking; OG = opinion giving; OP = opinion passing.

**Table 9:** Model fit index summary.

	χ^2^/d*f*	RMSEA	GFI	AGFI	CFI	NFI	TLI
Results of the research model fit indexes	1.487	0.040	0.912	0.904	0.949	0.932	0.939
Acceptable thresholds	≤3.00	≤0.070	≥0.900	≥0.900	≥0.900	≥0.900	≥0.900

**Table 10:** Results of regression weight.

Path			Estimate	Standardized Regression Estimate	S.E.	C.R.	*p*
Vigor	<---	AA_B	0.676	0.609	0.095	2.915	0.004 **
Absorption	<---	AA_B	0.529	0.562	0.096	2.380	0.017 *
Dedication	<---	AA_B	0.691	0.658	0.090	3.223	0.001 **
Vigor	<---	AA_S	0.529	0.579	0.058	5.686	***
Absorption	<---	AA_S	0.671	0.599	0.063	5.934	***
Dedication	<---	AA_S	0.550	0.538	0.055	4.583	***
Vigor	<---	CA_B	0.467	0.452	0.073	3.654	***
Absorption	<---	CA_B	0.519	0.456	0.077	3.779	***
Dedication	<---	CA_B	0.104	0.115	0.065	1.607	0.108
Vigor	<---	CA_S	0.413	0.417	0.227	3.573	***
Absorption	<---	CA_S	0.477	0.419	0.237	3.697	***
Dedication	<---	CA_S	0.538	0.524	0.186	2.889	0.004 **
OP	<---	AA_B	0.603	0.647	0.104	2.910	0.450
OG	<---	AA_B	0.528	0.485	0.107	4.925	***
OS	<---	AA_B	0.635	0.511	0.130	4.882	***
OP	<---	AA_S	0.487	0.606	0.081	6.041	***
OG	<---	AA_S	0.510	0.716	0.074	6.901	***
OS	<---	AA_S	0.326	0.400	0.081	4.040	0.123
OP	<---	CA_B	0.444	0.452	0.087	5.094	***
OG	<---	CA_B	0.350	0.402	0.075	4.660	***
OS	<---	CA_B	0.690	0.692	0.090	4.338	***
OP	<---	CA_S	0.936	0.906	0.353	4.630	***
OG	<---	CA_S	0.962	0.953	0.328	4.660	***
OS	<---	CA_S	0.985	0.811	0.335	4.428	***
OS	<---	Vigor	0.544	0.560	0.114	2.141	0.032 *
OG	<---	Vigor	0.443	0.474	0.096	1.480	0.139
OP	<---	Vigor	0.592	0.500	0.108	2.857	0.002 **
OS	<---	Absorption	0.382	0.435	0.103	3.704	***
OG	<---	Absorption	0.539	0.703	0.100	5.381	***
OP	<---	Absorption	0.518	0.600	0.108	4.808	***
OS	<---	Dedication	0.329	0.408	0.111	3.077	0 ***
OG	<---	Dedication	0.278	0.281	0.092	1.845	0.398
OP	<---	Dedication	0.656	0.552	0.107	2.527	0 ***

Note. * *p* < 0.05, ** *p* < 0.01, *** *p* < 0.001.

**Table 11:** Standardized total effects.

Variable	CA_S	CA_B	AA_S	AA_B
OP	0.300	0.288	0.398	0.362
OG	0.360	0.470	0.418	0.291
OS	0.305	0.428	0.449	0.331

**Table 12:** Standardized total effects—two tailed significance (BC).

Variable	CA_S	CA_B	AA_S	AA_B
OP	0.000	0.017	0.001	0.207
OG	0.000	0.039	0.000	0.012
OS	0.000	0.007	0.118	0.005

**Table 13:** Standardized direct effects.

Variable	CA_S	CA_B	AA_S	AA_B	Dedication	Absorption	Vigor
Dedication	0.130	0.136	0.236	0.258	0.000	0.000	0.000
Absorption	0.287	0.189	0.397	0.162	0.000	0.000	0.000
Vigor	0.146	0.231	0.288	0.276	0.000	0.000	0.000
OP	0.124	0.115	0.138	0.258	0.452	0.600	0.100
OG	0.219	0.256	0.199	0.162	0.381	0.703	0.174
OS	0.117	0.252	0.179	0.209	0.408	0.435	0.260

**Table 14:** Standardized indirect effects.

Variable	CA_S	CA_B	AA_S	AA_B
OP	0.176	0.173	0.260	0.104
OG	0.141	0.214	0.219	0.129
OS	0.188	0.176	0.270	0.122

**Table 15:** Standardized indirect effects—two tailed significance (BC).

Variable	CA_S	CA_B	AA_S	AA_B
OP	0.458	0.347	0.409	0.559
OG	0.360	0.307	0.331	0.554
OS	0.226	0.245	0.274	0.405

**Table 16:** Results of standardized regression weights.

Parameter			SE	SE-SE	Mean	Bias	SE-Bias
Vigor	<---	AA_B	0.033	0.003	0.638	0.029	0.004
Absorption	<---	AA_B	0.032	0.003	0.589	0.027	0.004
Dedication	<---	AA_B	0.044	0.003	0.688	0.030	0.005
Vigor	<---	AA_S	0.025	0.003	0.533	−0.046	0.004
Absorption	<---	AA_S	0.026	0.003	0.568	−0.031	0.004
Dedication	<---	AA_S	0.049	0.003	0.483	−0.055	0.005
Vigor	<---	CA_B	0.021	0.003	0.444	−0.008	0.004
Absorption	<---	CA_B	0.018	0.003	0.460	0.004	0.004
Dedication	<---	CA_B	0.033	0.003	0.115	0.000	0.004
Vigor	<---	CA_S	0.043	0.005	0.500	0.083	0.008
Absorption	<---	CA_S	0.025	0.005	0.493	0.074	0.006
Dedication	<---	CA_S	0.021	0.006	0.646	0.122	0.008
OP	<---	AA_B	0.041	0.012	0.702	0.055	0.017
OG	<---	AA_B	0.022	0.015	0.571	0.086	0.022
OS	<---	AA_B	0.052	0.008	0.543	0.032	0.011
OP	<---	AA_S	0.038	0.013	0.709	0.103	0.018
OG	<---	AA_S	0.043	0.015	0.870	0.154	0.022
OS	<---	AA_S	0.014	0.009	0.488	0.088	0.013
OP	<---	CA_B	0.036	0.010	0.515	0.063	0.014
OG	<---	CA_B	0.014	0.012	0.474	0.072	0.016
OS	<---	CA_B	0.025	0.007	0.741	0.049	0.009
OP	<---	CA_S	0.039	0.015	0.730	−0.176	0.021
OG	<---	CA_S	0.029	0.018	0.727	−0.226	0.025
OS	<---	CA_S	0.020	0.011	0.664	−0.147	0.015
OS	<---	Vigor	0.021	0.014	0.488	−0.072	0.020
OG	<---	Vigor	0.029	0.022	0.346	−0.128	0.031
OP	<---	Vigor	0.032	0.019	0.398	−0.102	0.027
OS	<---	Absorption	0.023	0.011	0.351	−0.084	0.015
OG	<---	Absorption	0.021	0.017	0.541	−0.162	0.024
OP	<---	Absorption	0.033	0.014	0.476	−0.124	0.020
OS	<---	Dedication	0.028	0.012	0.449	0.041	0.017
OG	<---	Dedication	0.027	0.020	0.304	0.023	0.029
OP	<---	Dedication	0.037	0.016	00.596	0.044	0.023
